# Nutrient profiles of commercially produced complementary foods available in Cambodia, Indonesia and the Philippines

**DOI:** 10.1017/S1368980022001483

**Published:** 2022-07-04

**Authors:** Eleonora Bassetti, Elizabeth Zehner, Susannah H Mayhew, Nadine Nasser, Anzélle Mulder, Jane Badham, Lara Sweet, Rachel Crossley, Alissa M Pries

**Affiliations:** 1 Helen Keller International, New York, NY, USA; 2 Department of Global Health and Development, London School of Hygiene and Tropical Medicine, London, UK; 3 Access to Nutrition Initiative, Utrecht, Netherlands; 4 JB Consultancy, Johannesburg, South Africa

**Keywords:** Nutrient profiling, Complementary foods, Infant and young child feeding, Malnutrition

## Abstract

**Objective:**

To assess the nutritional suitability of commercially produced complementary foods (CPCF) marketed in three South-East Asian contexts.

**Design:**

Based on label information declared on the products, nutrient composition and content of CPCF were assessed against the WHO Europe nutrient profile model (NPM). The proportion of CPCF that would require a ‘high sugar’ warning was also determined.

**Setting:**

Khsach Kandal district, Cambodia; Bandung City, Indonesia; and National Capital Region, Philippines.

**Participants:**

CPCF products purchased in Cambodia (*n* 68) and Philippines (*n* 211) in 2020, and Indonesia (*n* 211) in 2017.

**Results:**

Only 4·4 % of products in Cambodia, 10·0 % of products in Indonesia and 37·0 % of products in the Philippines fully complied with relevant WHO Europe NPM nutrient composition requirements. Sixteen per cent of CPCF in Cambodia, 27·0 % in Indonesia and 58·8 % in the Philippines contained total sugar content levels that would require a ‘high sugar’ warning.

**Conclusions:**

Most of the analysed CPCF were not nutritionally suitable to be promoted for older infants and young children based on their nutrient profiles, with many containing high levels of sugar and sodium. Therefore, it is crucial to introduce new policies, regulations and standards to limit the promotion of inappropriate CPCF in the South-East Asia region.

Early life is a critical window of opportunity to prevent all forms of malnutrition, including stunting, wasting, overweight/obesity and diet-related non-communicable diseases^(1,2)^. While breastmilk is sufficient for optimal infant growth until the age of 6 months, after this time infants’ nutritional needs increase beyond what exclusive breastfeeding can provide^(3)^. This period is an important time to focus on preventing growth faltering and future physical and neurocognitive limitations^(4)^. The introduction of safe, adequate and nutrient-dense complementary foods is necessary at this point, while breastfeeding continues to 2 years and beyond^(3)^. Older infants’ and young children’s (IYC) nutrient demands are very high and cannot be met if intake of nutrient-rich foods is low^(5)^.

Over the last decade, there has been substantial growth in the processed foods market in South-East Asia, including commercially produced complementary foods (CPCF), which have gained popularity due to their convenience and aggressive promotion^(6–8)^. Total sales of CPCF in the Philippines in 2020 were over US$ 30 million, having grown 10 % between 2015 and 2020^(9)^. Prior research also indicates that consumption of CPCF is increasingly prevalent among older IYC across the South-East Asia region, particularly in urban areas^(10,11)^. While nutrient-rich, home-prepared and locally available foods are preferable, commercial fortified food products added to diets of older IYC can improve micronutrient intake^(2)^. However, CPCF are heterogeneous in their nutritional quality. Some products may be of optimal nutrient composition, providing micronutrients that are typically missing in the diets of older IYC^(5,6,12)^, but others may be of concern due to high levels of added salt, sugar and other potentially harmful additives^(6)^. Unhealthy CPCF can also conceivably displace consumption of other nutritious foods and breastmilk, undermining optimal breastfeeding and complementary feeding practices^(7,13,14)^. Additionally, food preferences, tastes and habits established in early years of life can persist into later childhood and beyond^(2,15)^.

In 2016, the World Health Assembly (WHA) welcomed the WHO Guidance on Ending the Inappropriate Promotion of Foods for Infants and Young Children (WHA resolution 69.9)^(16)^. The WHO guidance called for restrictions on promotion of CPCF so that they do not interfere with breastfeeding, contribute to overweight and non-communicable diseases and create a dependency on commercial products or mislead caregivers (e.g. via inappropriate health and nutrition claims), whilst ensuring that products have high nutritional quality^(16)^. The WHO guidance suggested the development of nutrient profile models (NPM) to accompany decisions about which CPCF meet composition standards and ensure that only those that meet composition and labelling standards may be appropriately promoted^(16)^. Subsequently, in 2019, the WHO Regional Office for Europe created a NPM (hereafter referred to as WHO Europe NPM) to guide decisions on which products for children aged 6–36 months (older IYC) in the European region are suitable for promotion; in the WHO Europe NPM, a product must comply with both nutrient composition thresholds and labelling requirements to be suitable for promotion for older IYC^(17)^. The nutrient composition thresholds in the WHO Europe NPM are based on compositional requirements set out in regional and global guidance documents, including European Commission directives and Codex Alimentarius composition standards for CPCF^(17)^. In addition to classification of CPCF as suitable/unsuitable, the WHO Europe NPM also details a front-of-pack warning for products determined as having high sugar content.

As the double burden of malnutrition is a growing issue for IYC across the South-East Asia region, focus should be placed on the food environment, including the appropriateness of commercial complementary foods available on the market, to ensure optimal complementary feeding practices. Given their increasing demand in the South-East Asia region, it is essential that CPCF are of optimal nutritional quality^(17)^. The WHO Europe NPM has been validated for products in European markets; however, its applicability to other regions has not yet been evaluated. The application of such a NPM to products in other regions is a first step in enabling the use of nutrient profiling for CPCF in areas where consumption of these products is rising, such as the South-East Asia region, and where minimum standards for CPCF have yet to be established. This paper aims to assess the nutritional quality of CPCF available on the market in three South-East Asian contexts (Cambodia, Indonesia and the Philippines) to determine their nutritional suitability for older IYC. The objectives are (1) to determine the nutrient content of CPCF by capturing nutritional information declared on labels, including total sugar, added sugar, sodium and total fat, (2) to assess the nutritionally suitability of CPCF to be marketed to older IYC by applying the WHO Europe NPM, and (3) to determine the proportion of CPCF that would require a ‘high sugar’ warning based on the WHO Europe NPM.

## Methods

### Study design

This study involved a cross-sectional assessment of information declared on the labels of CPCF products available on the market in: Khsach Kandal district, Cambodia; Bandung City, Indonesia; and National Capital Region (NCR), Philippines. For this study, CPCF were defined as commercially produced foods specifically marketed as suitable for feeding children below 36 months of age, not including infant formula or other breastmilk substitutes. CPCF identified in the three locations had to meet at least one of the following criteria^(17)^: (1) recommended for introduction at an age of less than 3 years; (2) labelled with the words ‘baby’, ‘infant’, ‘toddler’, ‘young child’ or synonym; (3) labelled with an image of a child who appears to be younger than 3 years of age; or (4) in any other way presented as being suitable for children under the age of 3 years. Only food products, not beverage products, were included in this study.

### Store sampling and CPCF product identification

Store sampling for CPCF product purchasing applied the following procedures. In both Bandung City, Indonesia, and NCR, Philippines, store sampling followed WHO international procedures for the assessment of CPCF availability and promotion^(18)^. In Bandung City and NCR, forty-three stores were sampled in each location. Researchers purposively selected one small store per thirty-three randomly sampled public health facilities offering child health services in each city, resulting in thirty-three small stores visited in each location. Specifically, four small stores in closest proximity to each of the thirty-three health facilities were listed based on identification using Google Maps and Street View. Small stores included corner stores, neighbourhood cooperative grocery stores, minimarts and pharmacies. During data collection, these stores were visited in order of proximity and the first found to sell CPCF product was sampled for that facility. If a store not identified through Google Maps and Street View was found in closer proximity to the health facility and met study criteria, it was used instead. In addition, ten large retail outlets each in Bandung City and NCR were purposively sampled for their large variety of products that were assumed to be a representative sample of products available across the city. In each location, these ten large retail outlets were identified through store scoping and in consultation with local research staff familiar with the cities. Large retail stores included grocery stores/supermarkets, hypermarkets and baby stores. In NCR, five of the largest online retailer websites were also reviewed for CPCF products; however, no additional products were identified online that were not found in stores.

In Cambodia, CPCF products were identified and purchased in a peri-urban district, Khsach Kandal, outside Phnom Penh, using exhaustive sampling of stores within a subset of communes. Nine of eighteen communes in the district were selected for store sampling; these included the three most urban communes, the three more rural communes with the greatest population of children under 1 year of age and an additional three communes chosen by random selection. For each of the communes selected, researchers visited the village considered to be the most commercially developed and the village chief assisted researchers in identifying the main commercial road(s) in the village. The researchers went to the identified roads and located all stores selling baby goods (e.g. baby stores), medicines (e.g. pharmacies) or commercially produced foods (e.g. grocers, corner/convenience stores and kiosks) along the road(s) within the village boundaries. A total of 188 stores were visited, of which 13 % (*n* 25) sold CPCF.

Across all three locations, identification of CPCF within stores utilised the following procedures. All areas inside each store were surveyed (e.g. baby food section, milk section, baby supplies section and discount section) to identify all unique CPCF products for sale. The following criteria were used to determine whether CPCF products were unique or the same products, based on the assumption that these unique products may differ in nutrient content: (a) single serving and multi-serving packages of the same product were considered to be the same product; (b) different sizes of multi-serving packages were considered to be the same product; (c) bundles of single-serving sachets/packages were considered to be the same product; (d) products with the same name but different types of packaging (e.g. aluminium tin *v*. cardboard box) were considered to be the same product; (e) products with the same product name but different manufacturers (e.g. a local manufacturer *v*. an imported product with an international manufacturer) were considered to be unique products; (f) products that varied by brand/sub-brand were considered to be unique products; and (g) different flavours of the same product were considered to be unique products. CPCF products were purchased in 2017 in Bandung City, Indonesia, and in 2020 in Khsach Kandal, Cambodia and NCR, Philippines.

### Data management and analysis

After product purchase, the labels of the products were photographed/scanned. Only labels with information in English or the national language of the country were analysed. Label information in the national languages for Cambodia and Indonesia (Khmer and Bahasa Indonesia, respectively) were translated to English, while in the Philippines the label information was in both Filipino and English (as required by local regulations) and did not require translation. Data extraction was performed by entering all relevant information from the product labels into Microsoft Excel datasheets, including: ingredient list; declaration of nutrition information per serving and per 100 g (per ready-to-eat/powdered product); serving size; and nutrient content claims. Extracted data underwent a detailed error check in each location.

The labels of CPCF were assessed against the WHO Europe NPM to determine adherence to nutrient composition thresholds. The WHO Europe NPM contains two components to assess if a CPCF product is suitable for promotion – assessment of nutrient composition and assessment of labelling practices. As the aim of this study was to assess nutritional suitability of CPCF, only the nutrient composition component was used. First, product names and ingredients were reviewed, and CPCF products were placed in one of the sixteen categories proposed in WHO Europe CPCF NPM. After product categorisation, the ingredient list and nutritional content of products were cross-checked against category-specific nutrient/ingredients thresholds. These thresholds are typically applied to nutrient content per 100 kcal or per 100 g, but several thresholds are based on the presence of a nutrient/ingredient or another weight specification (e.g. per serving). Since the categories proposed in the WHO Europe NPM were created considering the products on the European market, they did not comprehensively reflect the types of CPCF on the market in the three South-East Asian contexts. Therefore, it was necessary to operate some adaptations. Specifically, thirteen products were dry powder products that were not applicable to category ‘1·1 Dry or instant cereal/starch’ because their main ingredient was dehydrated dairy/fruit. Though assessment of powdered products is intended to be based on non-reconstituted nutrient values, as the WHO Europe NPM is intended to cover foods ‘as sold’ rather than ‘as eaten’, these thirteen products were categorised under applicable puree categories and nutrient values assessed were based on reconstituted values as per manufacturers’ instructions. A considerable number of analysed products presented incomplete nutrient content information on the label. When product labels were missing nutrient content information for nutrient composition thresholds, these products were excluded from that specific nutrient assessment. A product was categorised as nutritionally suitable for older IYC if it achieved all category-specific nutrient thresholds.

The following listed ingredients were classed as added sugars/sweeteners: sugar or sucrose, dextrose, fructose, glucose, maltose, galactose, trehalose, (any) syrup, honey, malt extract, malted barley, molasses and juice/concentrate (other than lemon or lime juice, as they are not sweet tasting). The proportion of energy from sugar, fat and protein was determined using the Atwater factor system by multiplying the number of grams/100 g product of sugar, fat or protein by four, nine and four, respectively, and then dividing by the total energy content of the product (expressed in kcal/100 g). Where salt content, instead of sodium, was provided on the package, sodium content was estimated (total sodium = salt/2·5). Categorisation of products as fortified was based on review of ingredient lists for the addition of minerals/vitamins. The presence of nutrient content claims on products labels was determined by reviewing label information for statements/images that described the level of nutrient content^(19)^.

Analysis of the CPCF products’ performance in the WHO Europe NPM was conducted using a pre-designed Microsoft Excel spreadsheet developed by a team of researchers at Leeds University’s Nutritional Epidemiology Group, which serves as a WHO Collaborating Group. A user enters products’ ingredients and nutrient content based on the label declarations, and the spreadsheet contains automated calculations that evaluate each product against the WHO Europe NPM nutrient composition thresholds. Statistical analysis was conducted in Stata 14. Descriptive statistics were calculated and summarised using proportion and medians with minimum–maximum range for non-normally distributed data. Differences in proportions of products were tested using the Pearson chi-square test, with significance defined as *P* < 0·05.

## Results

A total of sixty-nine unique CPCF were purchased in Khsach Kandal district, Cambodia. One product did not provide any label information in either English or Khmer and was excluded, resulting in a final sample of sixty-eight CPCF products. In Bandung City, Indonesia, a total of 217 unique CPCF were purchased. Six of these did not meet the definition of CPCF used for this study and were excluded, resulting in a final sample of 211 products. In NCR, Philippines, a total of 211 unique CPCF were purchased. Characteristics of the CPCF identified in the three locations, including companies and brands, are provided in Supplemental Table 1. The majority of CPCF in Cambodia and Philippines were made by internationally headquartered companies, while almost half (44·5 %, *n* 94) of products purchased in Indonesia were made by national companies.

Instant cereals and ready-to-eat finger foods/snacks were the predominant categories of CPCF identified in both Khsach Kandal district, Cambodia (35·3 % and 54·4 %), and Bandung City, Indonesia (43·6 % and 33·6 %), while pureed foods/meals accounted for 57·3 % (*n* 121) of products identified in NCR, Philippines (Table 1). Only 4·4 % (*n* 3) products in Cambodia, 10·0 % (*n* 21) of products in Indonesia and 37·0 % (*n* 78) of products in the Philippines were found to be nutritionally suitable to be promoted for older IYC based on relevant WHO Europe NPM nutrient composition thresholds.

**Table 1:** WHO Europe NPM nutrient composition assessment of commercially produced complementary food products*

		Met all relevant nutrient thresholds		No added sugar/sweetener†		Low/no added fruit‡		Less than 15 % energy from sugar§		Met sodium threshold||		Met energy density threshold¶		Met protein threshold**		Met total fat threshold††	
Food category	*n*	%	*n*	%	*n*	%	*n*	%	*n*	%	*n*	%	*n*	%	*n*	%	*n*
Products identified in Khsach Kandal district, Cambodia (*n* 68)																	
Instant cereals																	
1·1 Dry or instant cereals/starch	24	12·5	3	20·8	5	91·7	22	NA		58·3	14	NA		87·5	21	87·5	21
Pureed foods/meals																	
2·1 Dairy-based desserts and cereal products	7	0·0	0	0·0	0	42·9	3	NA		0·0	0	100·0	7	100·0	7	100·0	7
Ready-to-eat finger foods/snacks																	
4·3 Snacks and finger foods	37	0·0	0	10·8	4	NA		48·7	18	21·6	8	NA		NA		75·7	28
Products identified in Bandung City, Indonesia (*n* 211)																	
Instant cereals																	
1·1 Dry or instant cereals/starch	92	7·6	7	31·5	29	94·6	87	NA		34·8	32	NA		92·4	85	94·6	87
Pureed foods/meals																	
2·1 Dairy-based desserts and cereal products	14	0·0	0	7·1	1	85·7	12	NA		100·0	14	78·6	11	21·4	3	78·6	11
2·2 Fruit puree	20	45·0	9	70·0	14	NA		NA		85·0	17	65·0	13	NA		100·0	2
2·3 Vegetable-only puree	2	50·0	1	100·0	2	100·0	2	NA		100·0	2	NA		NA		100·0	2
2·5 Pureed meal with cheese	2	50·0	1	100·0	2	100·0	2	NA		100·0	2	50·0	1	100·0	2	100·0	2
2·6 Pureed meal with meat/fish mentioned in product name	6	33·3	2	83·3	5	100·0	6	NA		50·0	3	66·7	4	83·3	5	100·0	6
2·7 Pureed meal with meat/fish not mentioned in product name	4	25·0	1	100·0	4	100·0	4	NA		75·0	3	25·0	1	100·0	4	75·0	3
Ready-to-eat finger foods/snacks																	
4·3 Snacks and finger foods	71	0·0	0	1·4	1	NA		9·9	7	42·3	30	NA		NA		62·0	44
Products identified in National Capital Region, Philippines (*n* 211)																	
Instant cereals																	
1·1 Dry or instant cereals/starch	35	54·3	19	71·4	25	82·9	29	NA		88·6	31	NA		100·0	35	100·0	35
Pureed foods/meals																	
2·1 Dairy-based desserts and cereal products	8	0·0	0	50·0	4	50·0	4	NA		100·0	8	100·0	8	87·5	7	100·0	8
2·2 Fruit puree	71	53·5	38	85·9	61	NA		NA		94·4	67	64·8	46	NA		100·0	71
2·3 Vegetable-only puree	8	75·0	6	100·0	8	100·0	8	NA		87·5	7	NA		NA		100·0	8
2·4 Vegetable puree with cereals	5	40·0	2	100·0	5	100·0	5	NA		100·0	5	40·0	2	NA		100·0	5
2·5 Pureed meal with cheese	5	80·0	4	100·0	5	100·0	5	NA		100·0	5	80·0	4	100·0	5	100·0	5
2·6 Pureed meal with meat/fish mentioned in product name	9	22·2	2	100·0	9	100·0	9	NA		77·8	7	66·7	6	22·2	2	100·0	9
2·7 Pureed meal with meat/fish not mentioned in product name	15	0·0	0	100·0	15	93·3	14	NA		66·7	10	33·3	5	26·7	4	100·0	15
3·1 Chunky meal with meat/fish/cheese	1	0·0	0	100·0	1	100·0	1	NA		100·0	1	NA		0·0	0	100·0	1
3·2 Chunky meal with vegetables	1	100·0	1	100·0	1	100·0	1	NA		100·0	1	NA		100·0	1	100·0	1
Ready-to-eat finger foods/snacks																	
4·1 Confectionary, sweet spreads, and chews/melts	4	0·0	0	25·0	1	NA		0·0	0	25·0	1	NA		NA		100·0	4
4·3 Snacks and finger foods	49	12·2	6	22·5	11	NA		53·1	26	42·9	21	NA		NA		91·8	45

* Values are presented as % (*n*); NA, not applicable based on category.

† The following were considered added sugar/sweetener: sugar, juice (except lemon/lime), sucrose, dextrose, fructose, glucose, maltose, galactose, trehalose, syrup, nectar, honey, malted barley, malt extract and molasses.

‡ Requirement definition per applicable category – 1·1: < 10 % by weight dried/powdered fruit; 2·1/2·5/2·6/2·7/2·8/3·1/3·2: ≤ 5 % by weight fruit puree; 2·3/2·4: no added fruit/fruit purée.

§ Applicable to category 4·3 only.

|| Threshold per applicable category – 1·1: sodium < 50 mg/100 kcal; 2·1/2·2/2·3/2·4/4·1/4·2/4·3: sodium < 50 mg/100 kcal and < 50 mg/100 g; 2·5: sodium < 100 mg/100 kcal and 100 mg/100 g; 2·6/2·7/2·8/3·1/3·2: sodium < 50 mg/100 kcal and < 50 mg/100 g (or < 100 mg/100 kcal and < 100 mg/100 g if cheese is listed in front-of-pack name).

¶ Threshold per applicable category – 2·1/2·2/2·4/2·5/2·6/2·7: energy density ≥ 60 kcal/100 g.

** Threshold per applicable category – 1·1: < 5·5 total protein g/100 kcal; 2·1: ≥ 2·2 total protein g/100 kcal; 2·5: ≥ 3 total protein g/100 kcal; 2·6: ≥ 4 total protein g/100 kcal from the named source and protein named as the first food(s) in the product name must be ≥ 10 % by weight of the total product; 2·7: ≥ 3 total protein g/100 kcal and protein source mentioned in the product name must be ≥ 8 % by weight of the total product; 2·8: ≥ 7 total protein g/100 kcal; 3·1: ≥ 4 total protein g/100 kcal and protein source mentioned in the product name must be ≥ 10 % by weight of the total product; 3·2: ≥ 3 total protein g/100 kcal.

†† Threshold per applicable category – 1·1/2·1/2·2/2·3/2·4/2·7/3·2/4·1/4·2/4·3: ≤ 4·5 g/100 kcal total fat; 2·5/2·6/2·8/3·1: ≤ 6 g/100 kcal total fat.

Of instant cereals, 12·5 % (*n* 3), 7·6 % (*n* 7) and 54·3 % (*n* 19) in Cambodia, Indonesia and the Philippines, respectively, were profiled as nutritionally suitable for promotion. The variance in performance of instant cereals was driven by variation in added/total sugar and sodium content in products; the presence of added sugar/sweeteners and failure to meet the sodium standard was more prevalent among instant cereals in Cambodia and Indonesia, as compared to the Philippines. Of pureed foods/meals, 0 %, 29·2 % (*n* 14) and 43·0 % (*n* 52) in Cambodia, Indonesia and the Philippines, respectively, were profiled as nutritionally suitable for promotion. The variance in performance of pureed foods/meals across the three locations was driven by the proportion of fruit-only and vegetable-only purees, which made up 65·3 % (*n* 79) and 45·8 % (*n* 22) of pureed foods/meals in the Philippines and Indonesia, as compared to 0 % in Cambodia. A greater proportion of fruit-only and vegetable-only purees (45–75 %) were profiled as nutritionally suitable, as compared to other puree categories. No dairy-based puree products in any of the three locations met their relevant WHO Europe NPM nutrient composition thresholds, primarily due to the presence of added sugar/sweeteners. The proportion of pureed foods/meals with meat/fish that were profiled as nutritionally suitable ranged from 0 to 33 % across the three locations, with failure to meet energy density and protein thresholds being the most common reasons for unsuitability. Ready-to-eat finger foods/snacks were consistently profiled as not nutritionally suitable for the promotion for older IYC across all three locations with 0 % in both Cambodia and Indonesia and only 11·3 % (*n* 6) in the Philippines meeting relevant WHO Europe NPM nutrient composition thresholds. This was primarily driven by the presence of added sugars/sweeteners and total sugar content in finger foods/snacks, but 78·4 %, 57·7 % and 57·1 % of finger foods/snacks in Cambodia, Indonesia and the Philippines, respectively, also failed to meet the sodium threshold. In Cambodia and Indonesia, a greater proportion of finger foods/snacks also failed to meet the total fat threshold, as compared to instant cereals or pureed foods/meals. Eighty or more per cent of all products in each of the three locations were able to achieve the nutrient composition threshold for total fat content.

Sixteen per cent (*n* 11) of CPCF products in Cambodia, 27·0 % (*n* 57) in Indonesia and 58·8 % (*n* 124) in the Philippines contained total sugar content levels that would require a ‘high sugar’ front-of-pack label warning based on WHO Europe NPM guidance (Table 2). This trend was driven primarily by ready-to-eat finger foods/snacks, such as rusks, biscuits and yogurt melts, where median total sugar content ranged from 12·8 to 55·8 g per 100 g of product across the three locations.

**Table 2:** Sugar warning and nutrient content of commercially produced complementary food products with relevant nutrient declarations*

		Requires ‘high sugar’ warning‡			Total sugar per 100 g (g)			Sodium per 100 g (mg)			Protein per 100 g (g)			Total fat per 100 g (g)	
Food category	*n* †	%	*n*	*n* †	Median	Minimum –Maximum	*n* †	Median	Minimum –Maximum	*n* †	Median	Minimum –Maximum	*n* †	Median	Minimum –Maximum
Products identified in Khsach Kandal district, Cambodia															
Instant cereals															
1·1 Dry or instant cereals/starch	9	0·0	0	9	9·2	0–15·0	21	161	5–325	21	12·5	5·2–16·0	21	7·5	0·8–10·0
Pureed foods/meals															
2·1 Dairy-based desserts and cereal products	0	–		0	–		0	–		7	3·3	3·0–3·5	7	2·8	2·5–3·0
Ready-to-eat finger foods/snacks															
4·3 Snacks and finger foods	29	37·9	11	29	12·8	0–66·7	30	279	0–500	33	5·1	0–13·3	33	0·8	0–28·6
Products identified in Bandung City, Indonesia															
Instant cereals															
1·1 Dry or instant cereals/starch	78	11·5	9	78	15·1	1·7–40·0	79	260	0–1256	87	12·0	4·2–22·9	87	7·0	0–14·6
Pureed foods/meals															
2·1 Dairy-based desserts and cereal products	11	45·5	5	11	5·7	1·3–11·0	14	13	2–29	14	1·0	0–4·5	14	2·7	1·2–2·9
2·2 Fruit puree	8	50·0	4	8	6·4	0·1–10·8	19	0	0–45	20	0	0–2·9	20	0	0–1·2
2·3 Vegetable-only puree	0	–		0	–		2	0	0–0	2	1·9	0·9–2·9	2	0·7	0–1·4
2·5 Pureed meal with cheese	2	0·0	0	2	0·7	0·5–0·8	2	52	42–63	2	2·7	2·5–2·8	2	2·1	1·2–2·9
2·6 Pureed meal with meat/fish mentioned in product name	2	0·0	0	2	1·2	0·8–1·5	6	30	18–64	6	2·9	2·5–4·8	6	1·6	0–2·9
2·7 Pureed meal with meat/fish not mentioned in product name	2	0·0	0	2	1·0	1·0–1·0	4	24	0–57	4	2·9	2·9–3·5	4	2·1	0–2·9
Ready-to-eat finger foods/snacks															
4·3 Snacks and finger foods	46	84·8	39	46	20·0	2·5–32·1	47	23	0–1595	47	5·3	0–10·0	47	7·5	0–32·5
Products identified in National Capital Region, Philippines															
Instant cereals															
1·1 Dry or instant cereals/starch	35	5·7	2	33	6·8	0–40·0	35	20	0–360	35	9·0	0·7–16·0	35	2·3	0–11·0
Pureed foods/meals															
2·1 Dairy-based desserts and cereal products	8	62·5	5	8	9·7	5·3–14·2	8	36	15–40	8	2·2	1·2–3·1	8	3·2	1·0–4·3
2·2 Fruit puree	71	100·0	71	71	10·4	4·2–16·4	71	4	0–80	71	0·8	0–1·7	71	0·2	0–2·2
2·3 Vegetable-only puree	8	50·0	4	8	3·0	1·8–8·0	8	8	0–31	8	1·3	0·6–2·4	8	0	0–1·5
2·4 Vegetable puree with cereals	5	60·0	3	5	2·8	1·7–7·8	5	10	7–27	5	1·9	1·5–2·7	5	0·4	0–1·0
2·5 Pureed meal with cheese	5	20·0	1	5	1·8	1·4–2·8	5	32	3–59	5	2·8	2·1–3·2	5	2·4	1·5–2·9
2·6 Pureed meal with meat/fish mentioned in product name	9	33·3	3	9	1·5	0·5–3·8	9	17	7–82	9	3·2	0·6–3·7	9	1·5	0·4–2·5
2·7 Pureed meal with meat/fish not mentioned in product name	15	46·7	7	15	2·4	0·9–38·5	15	27	11–45	15	2·9	1·8–3·5	15	1·2	0·7–2·2
3·1 Chunky meal with meat/fish/cheese	1	0·0	0	1	2·4	--	1	18	--	1	3·0	--	1	0·9	--
3·2 Chunky meal with vegetables	1	100·0	1	1	2·8	--	1	15	--	1	2·0	--	1	0·3	--
Ready-to-eat finger foods/snacks															
4·1 Confectionary, sweet spreads and chews/melts	4	100·0	4	4	55·8	4·0–57·1	4	211	14–286	4	16·6	1·0–28·6	4	0	0–11·8
4·3 Snacks and finger foods	49	46·9	23	47	12·9	0–57·1	49	71	0–929	49	4·6	0–40·0	49	6·0	0–28·6

* Values are presented as % (*n*) and median (minimum–maximum).

† Products without relevant nutrient content declarations on label are excluded.

‡ Front-of-pack ‘high sugar’ warning required if the percentage energy from total sugar content is greater than or equal to the standard for that product category – 1·1: 40 %; 2·1/2·2/2·3: 30 %; 2·4: 20 %; 2·5/2·6/2·7/3·1/3·2/4·1/4·3: 15 %.

**Fig. 1:**
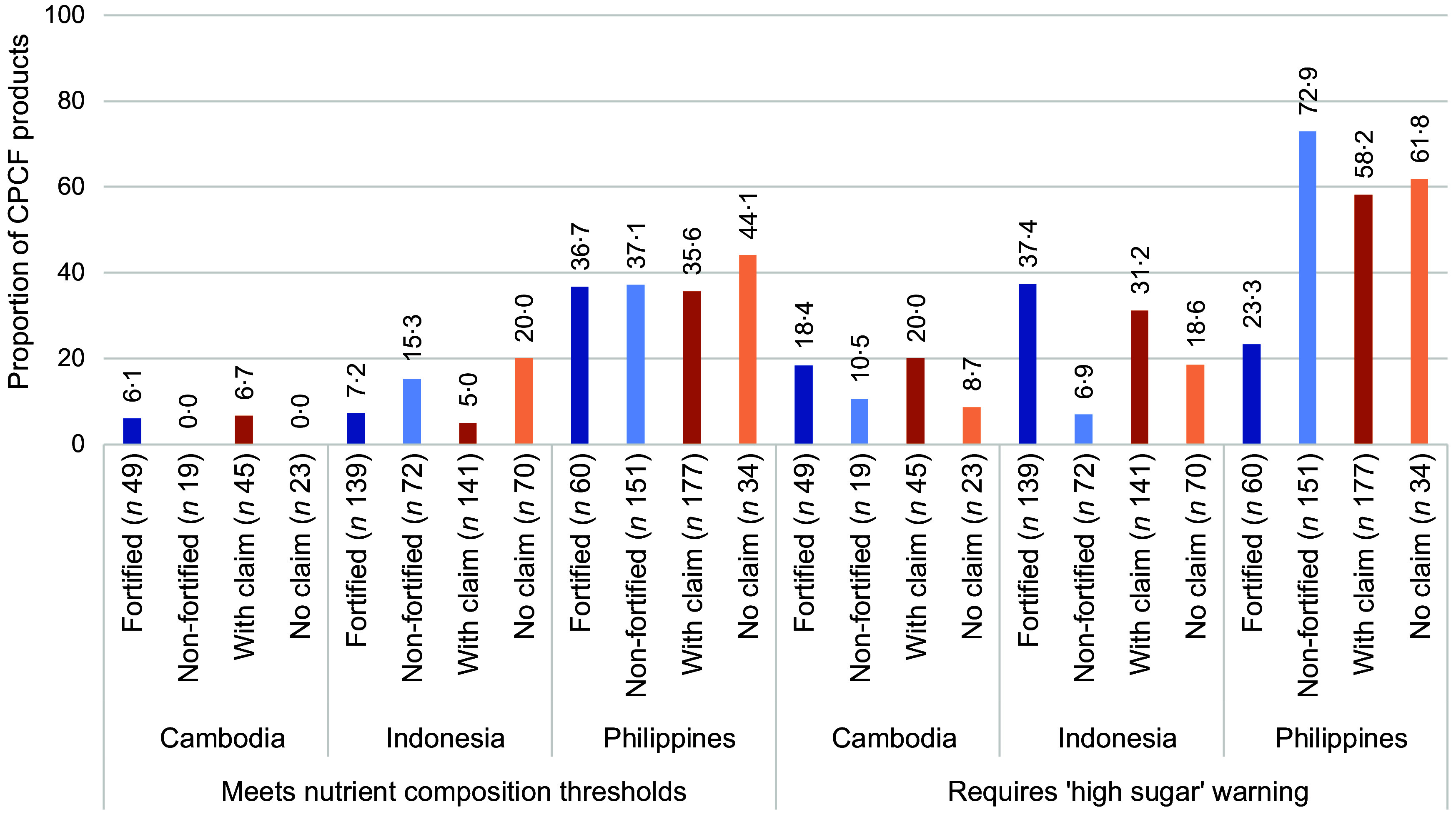
Nutrient profiling performance by fortification and nutrient content claim status. CPCF, commercially produced complementary foods.

Over two-thirds of CPCF products in Cambodia and Indonesia were fortified (72·1 % (*n* 49) and 65·9 % (*n* 139), respectively), while 28·4 % (*n* 60) of products in the Philippines were fortified. Nutrient content claims were present on 66·2 % (*n* 45) of CPCF purchased in Cambodia, 66·8 % (*n* 141) in Indonesia and 83·9 % (*n* 177) in the Philippines. The WHO Europe NPM performance of fortified *v*. non-fortified products and products with nutrient content claims *v*. no claims varied across the three locations (Fig. 1). In Cambodia, there was no statistically significant difference in products’ ability to meet nutrient composition thresholds of the WHO Europe NPM nor in products that would require a ‘high sugar’ front-of-pack warning. A similar trend was found in the Philippines, with the exception that a lower proportion of fortified products were required to carry a ‘high sugar’ front-of-pack warning as compared to non-fortified products (*P* < 0·001). While in Indonesia, a greater proportion of non-fortified products and products without nutrient content claims met all nutrient composition thresholds as compared to fortified products (*P* < 0·001) and products with claims (*P* < 0·001). In addition, a greater proportion of fortified products and products with nutrient content claims required ‘high sugar’ front-of-pack warnings as compared to non-fortified or products without claims (*P* < 0·001 and *P* = 0·052, respectively).

## Discussion

This study assessed the nutritional suitability of CPCF marketed in: Khsach Kandal district, Cambodia Bandung City, Indonesia; and NCR, Philippines. Nutrient profiles were evaluated against the nutrient content component of the WHO Europe NPM for CPCF. To our knowledge, this is the first study to conduct nutrient profiling of CPCF across the South-East Asia region and the first study to apply the WHO Europe NPM in another region. Ninety-six per cent of products in Cambodia, 90·0 % in Indonesia and 63·0 % in the Philippines were classified by the WHO Europe NPM as not suitable to be promoted to older IYC based on their nutrient content. Median sugar content was greatest among snack/finger food products, at 12·8 g and 20·0 g per 100 g product in Cambodia and Indonesia, respectively, and among confectionary items in the Philippines, at 55·8 g per 100 g product.

Most of the CPCF products were not compliant with WHO Europe NPM recommendations due to the presence of added sugars and/or excessive sodium content. These findings are consistent with recent studies conducted in Europe that also used the WHO Europe NPM^(20,21)^ and found prevalent use of added sugars in CPCF. High sugar content in CPCF has also been found among products in other countries using different evaluation approaches. A study that evaluated the impact of Chilean front-of-package warning label found that 40 % of formulas and foods for older IYC available in Chile had high sugar content that would warrant a front-of-package warning^(22)^. Likewise, 45 % of CPCF available in the USA were classified as having ‘high’ sugar content when evaluated in light of American Heart Association recommendations, with over 20 % of their energy content derived from sugar^(23)^. This goes against recommendations of several international health agencies and associations to limit free sugars and avoid added sugars in foods for older IYC. The WHO recommends limiting free sugar intake to below 10 % of total energy intake for adults and suggests a further reduction to below 5 %^(24)^. However, for children below 2 years of age, the European Society for Paediatric Gastroenterology Hepatology and Nutrition (ESPGHAN)^(25)^, the American Heart Association recommends to avoid added sugars^(26)^ and the WHO Europe NPM recommends avoiding any added sugars or sweetening agents for children below '36 months of age. In addition, excessive sodium content was particularly problematic in Cambodia and Indonesia, where only one-third and one-half of products, respectively, fell below the WHO Europe NPM sodium standard. While sodium content standards for CPCF do not exist in Cambodia, in Indonesia, the sodium standard for CPCF is below 100 mg/100 kcal^(27)^, twice as high as the WHO Europe NPM standard (50 mg/100 kcal). The high sugar and salt content of CPCF analysed in this study is extremely concerning. As the consumption of CPCF in South-East Asia is rising, older IYC might be increasingly exposed to unhealthy products that contribute to their daily sugar and sodium intakes and may displace the consumption of more nutritious whole foods. High sugar intake is linked with the development of dental caries^(28)^, weight gain and increased risk of non-communicable diseases^(29)^, and high salt intake in early life is correlated with high blood pressure in childhood^(30)^. Moreover, infants are born with a preference for sweet and salty tastes^(15)^, and exposure to these tastes early in life can establish long-lasting taste preferences and unhealthy dietary patterns^(15,31)^. Therefore, it is crucial to minimise added sugar and salt content in the diet of older IYC and to diversify exposure to different flavours to ensure the acceptability of a wider range of nutritious foods later in life^(32)^. A growing amount of data suggests that promotion of unhealthy CPCF may jeopardise appropriate nutrition during the complementary feeding period^(2)^ and later in life^(33)^. Consequently, prohibiting added sugars and limiting salt and total sugars in CPCF should be a policy priority for national governments, as well as among manufacturers, to ensure that older IYC are not exposed to products that are unnecessarily sweet or salty on a regular basis.

Despite widespread recognition of the need to reduce sugar intake in children^(2,24)^, progress in lowering the sugar content of products marketed to IYC has been insufficient. It is critical to advocate a new approach to sugar reduction that focuses on avoiding added sugars and sweetness intensity^(34)^. While the recommendation to prohibit added sugars in CPCF may be considered challenging to implement, it could be an effective strategy^(35,36)^ to reduce sugar intake of older IYC at the population level in South-East Asia, given their increasing consumption of CPCF. The food industry may be concerned that consumers will find reformulated low-sugar products less acceptable; however, a recent study showed that a low amount of sugar in CPCF instant cereals did not compromise their acceptability^(37)^. National CPCF nutrient composition standards are typically guided by Codex Alimentarius (Codex) standards in the South-East Asia region. However, Codex does not have a compositional requirement for total sugar or added/free sugar. This gap in Codex guidance may therefore be exploited by the food industry in the production and marketing of CPCF and must be addressed with stricter standards. The WHO Guidance on Ending the Inappropriate Promotion of Foods for Infants and Young Children in recommendation 3 specifies that ‘Codex standards and guidelines should be updated, and additional guidelines developed in line with WHO’s guidance to ensure that products are appropriate for IYC, with a particular focus on avoiding the addition of free sugars and salt’^(16)^. As the amount of added sugar is not routinely indicated on CPCF packages and thus difficult to monitor in contexts within South-east Asia, it would be more practical to prohibit added sugars rather than setting a limit on the authorised amount of added sugar.

Over half of the CPCF identified in Cambodia, one-third in Indonesia and one-quarter in Philippines were snack/finger foods and confectionary items. The CPCF snack/finger food products identified in this study included sweet/savoury puffs, biscuits/cookies, yogurt candies and instant noodles, which are common types of snack food products for the general population. There is growing evidence that older IYC consume commercial snack products during the critical complementary feeding period^(7,38)^. A survey conducted in Phnom Penh in 2014 found that 55 % of children 6–23 months of age had consumed commercially produced snack foods in the previous 24 h^(39)^, and a 2018 survey found such consumption among 82 % of children 6–35 months of age in Bandung City, Indonesia^(10)^. The ubiquitous presence of snack food products on the market may normalise older IYC snacking on commercial foods and encourage undesirable habits throughout childhood^(17)^. Furthermore, the CPCF snack/finger foods identified in this study had particularly poor nutrient profiles, with none in Cambodia and Indonesia and only 11 % in Philippines found to be nutritionally suitable to be promoted for older IYC. These products contained excessive sugar, with 38–85 % requiring a ‘high sugar’ warning across the three locations. Median total sugar content for these products was 13–56 g per 100 g across the three locations; in comparison, total sugar content per 100 g of commercial biscuits in the United Kingdom ranges from 17 to 45 g^(40)^. It is of great concern that a substantial portion of the CPCF product landscape in these South-East Asian countries is commercial snack food products high in sugar/sodium and that they are promoted as products suitable for older IYC when their nutrient profiles indicate they are not appropriate for this age group. Overconsumption of energy-dense, nutrient-poor snack foods products can contribute to undernutrition and micronutrient deficiencies by displacing consumption of whole foods rich in essential nutrients^(13)^, as well as overweight and obesity^(41)^, exacerbating the double burden of malnutrition already prevalent in the region.

The use of nutrient content claims on CPCF products in Cambodia, Indonesia and the Philippines was widespread. Nutrient content claims were present on 66 %, 67 % and 84 % of labels, respectively. Nutrient content claims are likely to attract caregivers, creating a healthy ‘halo effect’, establishing brand -loyalty and idealising the product^(42)^, which can put home-made foods at a disadvantage^(17)^. Such claims can be misleading, especially when placed on products with high levels of nutrients of public health concern^(42)^. In this study, although many CPCF claimed to offer nutrient content benefits, particularly the presence of vitamins and minerals, most of them contained concerning levels of sugar and sodium. In Indonesia and the Philippines, fewer products with nutrient content claims met all WHO Europe NPM nutrient composition thresholds than those without nutrient content claims, and in Cambodia and Indonesia a greater proportion of products with such claims were required to carry a ‘high sugar warning’ as compared to products with no nutrient content claim. While the Philippines prohibits the presence of nutrient content claims on CPCF products^(43)^ and Indonesia prohibits such claims on products promoted for infants below 12 months^(44)^, findings from this study indicate that stronger enforcement of national regulations and company compliance are urgently needed to protect older IYC diets from misleading and confusing marketing and promotional tactics.

Introducing new national policies, regulations and standards to address unhealthy food environments and to limit the promotion of inappropriate CPCF will simultaneously tackle malnutrition in all its forms. The balance between the need for additional micronutrients in the diet of older IYC and the potential harm from consuming CPCF that additionally contain high sugar and sodium content poses many challenges. Implementing an adapted version of the WHO Europe NPM in South-East Asia, which aligns with national dietary guidelines, could be a valuable way to assess CPCF sold in the region. An adapted NPM could include the evaluation of relevant micronutrient content, such as iron, zinc and calcium, to ensure that CPCF provide adequate amounts of micronutrients of public health concern. Moreover, enforcing a standardised front-of-package labelling system could be a cost-effective intervention, since it would encourage healthier food choices, help caregivers in making informed purchasing decisions^(17,45,46)^ and urge manufacturers to reformulate unhealthy CPCF^(47,48)^. Further research is needed to define what standardised front-of-package labelling system would be most effective and acceptable in assisting caregivers in South-East Asia contexts to make informed CPCF purchases.

This study has several limitations. Firstly, the study was restricted to the information provided on product labels. Not all nutrients appeared on all labels, making it challenging to determine whether the product met all relevant nutrient standards. When a product was missing data relative to a nutrient, it was excluded from assessment for that specific nutrient. This could have led to an underestimation of the products meeting WHO Europe NPM criteria. Secondly, the analysis relied on manufacturers’ reported content, rather than independent laboratory analyses. Thus, actual nutrient content may be higher or lower than that declared on the label. Third, the study focused on the nutritional quality of CPCF and did not assess the overall quality of the diet of older IYC. Nonetheless, the analysis highlights the concerning amount of total sugar and sodium contained in CPCF, which could potentially contribute to sugar and sodium intake in the diet of older IYC consuming these products. Further research is needed to determine CPCF’s contributions of these problematic nutrients to diets of older IYC in South-East Asia. Finally, the WHO Europe NPM was developed for the European region. It was not designed specifically for the diet/nutritional status of South-East Asian older IYC and does not consider the importance of micronutrient content in CPCF for this population.

## Conclusion

This study contributes evidence to a currently limited field of research investigating the nutrient profiles and nutrient content of CPCF. The findings reveal that many products provide excess nutrients of public health concern, such as sugar and sodium, and do not comply with WHO recommendations for nutrient composition for diets of older IYC. As food companies increase their market penetration in low- and middle-income countries, CPCF will become more abundant and even more pervasively promoted. To reduce the availability of CPCF high in sugar and salt, further restrictions on food promotion are therefore necessary in the South-East region to improve children’s diets, foster healthy food environments and prevent malnutrition in all its forms. Stricter and more comprehensive standards related to labelling practices should be enacted and enforced to provide clearer and more comprehensive label information and to prohibit deceptive nutrient content claims on products that are not nutritionally suitable for this vulnerable age group. To address the increasing prevalence of the double burden of malnutrition in South-East Asia, regulations and enforcement to hold manufacturers accountable are urgently needed.

## Supplementary material

For supplementary material accompanying this paper visit https://doi.org/10.1017/S1368980022001483. click here to view supplementary material

## References

[1] Wells JC , Sawaya AL , Wibaek R et al. (2020) The double burden of malnutrition: aetiological pathways and consequences for health. Lancet 395, 75–88. 31852605 10.1016/S0140-6736(19)32472-9 PMC7613491

[2] United Nations Children’s Fund (UNICEF) (2020) Improving Young Children’s Diets During The Complementary Feeding Period. New York: UNICEF Programming Guidance.

[3] WHO & UNICEF (2003) Global Strategy for Infant and Young Child Feeding. Geneva: WHO.

[4] de Onis M & Branca F (2016) Childhood stunting: a global perspective. Matern Child Nutr 12, 12–26. 27187907 10.1111/mcn.12231 PMC5084763

[5] Dewey KG (2013) The challenge of meeting nutrient needs of infants and young children during the period of complementary feeding: an evolutionary perspective. J Nutr 143, 2050–2054. 24132575 10.3945/jn.113.182527 PMC3827643

[6] Zehner E , Champeny M & Huffman SL (2019) Marketing and infant and young child feeding in rapidly evolving food environments. Matern Child Nutr 15, 1–6. 10.1111/mcn.12810 PMC6618061 31225711

[7] Pries AM , Filteau S & Ferguson EL (2019) Snack food and beverage consumption and young child nutrition in low- and middle-income countries: a systematic review. Matern Child Nutr 15, e12729. 31225715 10.1111/mcn.12729 PMC6618154

[8] Sievert K , Lawrence M , Naika A et al. (2019) Processed foods and nutrition transition in the Pacific: regional trends, patterns and food system drivers. Nutrients 11, 1328. 31200513 10.3390/nu11061328 PMC6628317

[9] Access to Nutrition Initiative (2021) Landscape Study: the Philippines: Complementary Feeding and the Role of Commercially Produced Complementary Foods in Young Children’s Diets. https://accesstonutrition.org/app/uploads/2021/05/ATNI_PH-CPCF-landscape-study.pdf (accessed October 2021).

[10] Green M , Hadihardjono DN , Pries AM et al. (2019) High proportions of children under 3 years of age consume commercially produced snack foods and sugar – sweetened beverages in Bandung City, Indonesia. Matern Child Nutr 15, e12764. 31225706 10.1111/mcn.12764 PMC6619027

[11] Jacquier EF , Angeles-Agdeppa I , Lenighan YM et al. (2020) Complementary feeding patterns of Filipino infants and toddlers lack diversity, especially among children from poor households. BMC Nutr 6, 51. 33117553 10.1186/s40795-020-00376-1 PMC7586690

[12] Swanepoel E , Havemann-Nel L , Rothman M et al. (2019) Contribution of commercial infant products and fortified staplefoods to nutrient intake at ages 6, 12, and 18 months in acohort of children from a low socio-economic community in South Africa. Matern Child Nutr 5, e12674. 10.1111/mcn.12674 PMC7198934 30216697

[13] Pries AM , Rehman AM , Filteau S et al. (2019) Unhealthy snack food and beverage consumption is associated with lower dietary adequacy and length-for-age z -scores among 12–23-month-olds in Kathmandu Valley, Nepal. J Nutr 149, 1843–1851. 31309223 10.1093/jn/nxz140 PMC6768809

[14] Feeley AB , Ndeye Coly A , Sy Gueye NY et al. (2016) Promotion and consumption of commercially produced foods among children: situation analysis in an urban setting in Senegal. Matern Child Nutr 12, 64–76. 10.1111/mcn.12304 PMC5071683 27061957

[15] De Cosmi V , Scaglioni S & Agostoni C (2017) Early taste experiences and later food choices. Nutrients 9, 107. 28165384 10.3390/nu9020107 PMC5331538

[16] World Health Organization (2017) Guidance on Ending the Inappropriate Promotion of Foods for Infants and Young Children: Implementation Manual. Geneva: WHO.

[17] World Health Organization (2019) Ending Inappropriate Promotion of Commercially Available Complementary Foods for Infants and Young Children between 6 and 36 Months in Europe. http://www.euro.who.int/__data/assets/pdf_file/0004/406453/Ending_Final_3June2019.pdf?ua=1 (accessed October 2021).

[18] World Health Organization & United Nations Children’s Fund (UNICEF) (2017) NetCode Toolkit. Monitoring the Marketing of Breast-Milk Substitutes: Protocol for Periodic Assessments. Geneva: WHO.

[19] Codex Alimentarius (2013) Guidelines for Use of Nutrition and Health Claims – CAC/GL 23–1997. https://www.fao.org/ag/humannutrition/32444-09f5545b8abe9a0c3baf01a4502ac36e4.pdf (accessed October 2021).

[20] Hutchinson J , Rippin H , Threapleton D et al. (2021) High sugar content of European commercial baby foods and proposed updates to existing recommendations. Matern Child Nutr 17, e13020. 32862552 10.1111/mcn.13020 PMC7729710

[21] Pace L , Bica M , Williams J et al. (2020) High levels of sugar and salt in commercial baby foods in Malta: results from a pilot study using the world health organization draft nutrient profile model. Malta Med J 32, 59–76.

[22] Scarpelli D , Fernandes A , Osiac L et al. (2020) Changes in nutrient declaration after the food labeling and advertising law in Chile: a longitudinal approach. Nutrients 12, 2371. 32784370 10.3390/nu12082371 PMC7468860

[23] Elliott C & Conlon M (2014) Packaged baby and toddler foods: questions of sugar and sodium. Pediatr Obes 10, 149–155. 24756975 10.1111/j.2047-6310.2014.223.x

[24] World Health Organization (2015) Guideline: Sugars Intake for Adults and Children. Geneva: World Health Organization. 25905159

[25] Fidler Mis N , Braegger C , Bronsky J et al. (2017) Sugar in infants, children and adolescents: a position paper of the European society for paediatric gastroenterology, hepatology and nutrition committee on nutrition. J Pediatr Gastroenterol Nutr 65, 681–696. 28922262 10.1097/MPG.0000000000001733

[26] Vos MB , Kaar JL , Welsh JA et al. (2017) Added sugars and cardiovascular disease risk in children: a scientific statement from the American Heart Association. Circulation 135, e1017–e1034. 27550974 10.1161/CIR.0000000000000439 PMC5365373

[27] Peraturan Badan Pengawas Obat Dan Makanan (PerBPOM) (2018) Nomor 1 tahun 2018, Pengawasan pangan olahan untuk keperluan gizi khusus. Republik Indonesia (Number 1 of 2018, Supervision of processed food for special nutritional purposes). https://standarpangan.pom.go.id/dokumen/peraturan/2018/PerBPOM_1_Tahun_2018_PKGK_Join.pdf (accessed October 2021).

[28] Ruottinen S , Karjalainen S , Pienihäkkinen K et al. (2004) Sucrose intake since infancy and dental health in 10-year-old children. Caries Res 38, 142–148. 14767171 10.1159/000075938

[29] Breda J , Jewell J & Keller A (2019) The importance of the World Health Organization sugar guidelines for dental health and obesity prevention. Caries Res 53, 149–152. 30086553 10.1159/000491556 PMC6425811

[30] Genovesi S , Giussani M , Orlando A et al. (2021) Salt and sugar: two enemies of healthy blood pressure in children. Nutrients 13, 697. 33671538 10.3390/nu13020697 PMC7927006

[31] Liem DG (2017) Infants’ and children’s salt taste perception and liking: a review. Nutrients 9, 1011. 28902163 10.3390/nu9091011 PMC5622771

[32] Hawkes C , Smith TG , Jewell J et al. (2015) Smart food policies for obesity prevention. Lancet 385, 2410–2421. 25703109 10.1016/S0140-6736(14)61745-1

[33] Foterek K , Buyken AE , Bolzenius K et al. (2016) Commercial complementary food consumption is prospectively associated with added sugar intake in childhood. Br J Nutr 115, 2067–2074. 27079145 10.1017/S0007114516001367

[34] Velázquez AL , Vidal L , Varela P et al. (2021) Sugar reduction in products targeted at children: why are we not there yet? J Sens Stud 36, e12666.

[35] Hashem KM , He FJ & Macgregor GA (2019) Effects of product reformulation on sugar intake and health-a systematic review and meta-analysis. Nutr Rev 77, 181–196. 30624760 10.1093/nutrit/nuy015

[36] Muth MK , Karns SA , Mancino L et al. (2019) How much can product reformulation improve diet quality in households with children and adolescents? Nutrients 11, 618. 30875736 10.3390/nu11030618 PMC6470779

[37] Sanchez-Siles LM , Bernal MJ , Gil D et al. (2020) Are sugar-reduced and whole grain infant cereals sensorially accepted at weaning? A randomized controlled cross-over trial. Nutrients 12, 1883. 32599738 10.3390/nu12061883 PMC7353261

[38] Pries AM , Huffman SL , Champeny M et al. (2017) Consumption of commercially produced snack foods and sugar-sweetened beverages during the complementary feeding period in four African and Asian urban contexts. Matern Child Nutr 13, e12412. 29032629 10.1111/mcn.12412 PMC6865897

[39] Pries A , Huffman S , Mengkheang K et al. (2016) High use of commercial food products among infants and young children and promotions for these products in Cambodia. Matern Child Nutr 12, 52–63. 27061956 10.1111/mcn.12270 PMC5021124

[40] Public Health England (2021) McCance and Widdowson’s Composition of Foods Integrated Dataset. https://www.gov.uk/government/publications/composition-of-foods-integrated-dataset-cofid (accessed December 2021).

[41] Black RE , Victora CG , Walker SP et al. (2013) Maternal and child undernutrition and overweight in low-income and middle-income countries. Lancet 382, 427–451. 23746772 10.1016/S0140-6736(13)60937-X

[42] Harris JL , Thompson JM , Schwartz MB et al. (2011) Nutrition-related claims on children’s cereals: what do they mean to parents and do they influence willingness to buy? Public Health Nutr 14, 2207–2212. 21806872 10.1017/S1368980011001741

[43] Republic of the Philippines Department of Health (2014) Rules and Regulations for the Labelling of Pre-packaged Food Products Distributed in the Philippines. Administrative Order No. 2014–0030. https://www.fda.gov.ph/wp-content/uploads/2021/03/Administrative-Order-No.-2014-0030.pdf (accessed December 2021).

[44] Peraturan Kepala Badan Pengawas Obat Dan Makanan (PKBPOM) (2016) Nomor 13 tahun 2016, Pengawasan klaim pada label dan iklan pangan olahan (Number 13 of 2016, Supervision of claims on processed food labels and advertisements). https://standarpangan.pom.go.id/dokumen/peraturan/2016/PerKa_BPOM_No_13_Tahun_2016_tentang_Klaim_pada_Label_dan_Iklan_Pangan_Olahan.pdf (accessed October 2021).

[45] Croker H , Packer J , Russell SJ et al. (2020) Front of pack nutritional labelling schemes: a systematic review and meta-analysis of recent evidence relating to objectively measured consumption and purchasing. J Hum Nutr Diet 33, 518–537. 32364292 10.1111/jhn.12758

[46] World Health Organization (2017) Report of the Commission on Ending Childhood Obesity: Implementation Plan. https://apps.who.int/gb/ebwha/pdf_files/WHA70/A70_31-en.pdf (accessed December 2021).

[47] Ni Mhurchu C , Eyles H & Choi YH (2017) Effects of a voluntary front-of-pack nutrition labelling system on packaged food reformulation: the health star rating system in New Zealand. Nutrients 9, 918. 28829380 10.3390/nu9080918 PMC5579711

[48] Roberto CA , Ng SW , Ganderats-Fuentes M et al. (2021) The influence of front-of-package nutrition labeling on consumer behavior and product reformulation. Annu Rev Nutr 41, 529–550. 34339293 10.1146/annurev-nutr-111120-094932

