# Supplementary material for: Nutrient profiles of commercially produced complementary foods available in Cambodia, Indonesia and the Philippines
Source: Public Health Nutr. 2022 Jul 4;25(10):2720–30. doi: 10.1017/S1368980022001483 (PMC9991791; doi:10.1017/S1368980022001483)
Supplement: Supplementary file 1 [file S1368980022001483sup001.docx]

| Supplemental Table 1. Commercially produced complementary food product characteristics, by location | | | | |
| --- | --- | --- | --- | --- |
| Company | Company headquarters location | Brand | Product category | % (n)^2^ |
| Products identified in Khsach Kandal district, Cambodia (n=68) | | | | |
| Gerber Products Co. | USA | Gerber | Snacks and finger foods | 14.7 (10) |
| Guangzhou Yong Want Foods Ltd. | China | Want-Want | Snacks and finger foods | 11.8 (8) |
|  |  | Hot-Kid | Snacks and finger foods | 2.9 (2) |
| Kalbe | Indonesia | Milna | Dry or instant cereals/starch (n=5); Snacks and finger foods (n=3) | 11.8 (8) |
| Nestlé | Switzerland | Cerelac | Dry or instant cereals/starch | 10.3 (7) |
| Angkor Dairy Products Co. Ltd. | Cambodia | Angkormilk | Pureed foods | 8.8 (6) |
| Healthy Foods Co. Ltd. | Thailand | Apple Monkey | Snacks and finger foods | 7.4 (5) |
| Dana Dairy Group Ltd. | Switzerland | Dana Milk | Dry or instant cereals/starch | 5.9 (4) |
| Indofood CBP Sukses Makmur | Indonesia | Promina | Snacks and finger foods | 5.9 (4) |
| Namchow Ltd. | Thailand | Namchow | Snacks and finger foods | 4.4 (3) |
| Peachy Village Co. Ltd. | Thailand | Peachy | Snacks and finger foods | 4.4 (3) |
| Regilait | France | France Lait | Dry or instant cereals/starch | 4.4 (3) |
| *company not provided | --- | Little Bio Organic | Dry or instant cereals/starch | 2.9 (2) |
| International Dairy Product JSC | Vietnam | Love’in Farm | Pureed foods | 1.5 (1) |
| Happy Family Brands | USA | Organics Happy Baby | Snacks and finger foods | 1.5 (1) |
| Mondelez Kinh Do JSC | Vietnam | AFC Nutrition | Snacks and finger foods | 1.5 (1) |
| Products identified in Bandung City, Indonesia (n=211) | | | | |
| Indofood CBP Sukses Makmur | Indonesia | Promina | Dry or instant cereals/starch (n=16); Pureed foods (n=2); Snacks and finger foods (n=7) | 11.8 (25) |
|  | Indonesia | SUN | Dry or instant cereals/starch (n=17); Pureed foods (n=2); Snacks and finger foods (n=4) | 10.9 (23) |
| H.J. Heinz Company | USA | Heinz | Dry or instant cereals/starch (n=7); Pureed foods (n=25); Snacks and finger foods (n=5) | 17.5 (37) |
|  | USA | Farley’s | Snacks and finger foods | 1.4 (3) |
| Kalbe | Indonesia | Milna | Dry or instant cereals/starch (n=18); Pureed foods (n=4); Snacks and finger foods (n=8) | 14.2 (30) |
| Nestlé | Switzerland | Cerelac | Dry or instant cereals/starch | 6.2 (13) |
|  | Switzerland | Gerber | Dry or instant cereals/starch (n=4); Snacks and finger foods (n=7) | 5.2 (11) |
| Peachy Village Co. Ltd. | Thailand | Peachy | Pureed foods (n=11); Snacks and finger foods (n=4) | 7.1 (15) |
| *company not provided | --- | Yummy Bites | Snacks and finger foods | 5.2 (11) |
| Gasol Pertanian Organik | Indonesia | Gasol | Dry or instant cereals/starch (n=8); Pureed foods (n=1) | 4.2 (9) |
| Hero Spain | Spain | Organix | Dry or instant cereals/starch (n=4); Snacks and finger foods (n=3) | 3.3 (7) |
| Happy Family Brands | USA | Organics Happy Baby | Snacks and finger foods | 2.4 (5) |
| Oyatsu Company Ltd. | Japan | Baby Star | Snacks and finger foods | 1.9 (4) |
| Bionic Natura | Indonesia | Bionic Farm Organic | Dry or instant cereals/starch | 1.9 (4) |
| *company not provided | --- | Bebenice | Snacks and finger foods | 1.9 (4) |
| EMPRO Singapore PTE Ltd. | Singapore | Baby Choice | Snacks and finger foods | 1.4 (3) |
| Vitagermine SAS | France | Babynat | Pureed foods | 0.9 (2) |
|  | France | Babybio | Pureed foods | 0.5 (1) |
| Negro Brand | Indonesia | Negro-Brand | Dry or instant cereals/starch | 0.5 (1) |
| Morinaga & Company | Japan | Morinaga | Snacks and finger foods | 0.5 (1) |
| Sehati | Indonesia | Sehati | Snacks and finger foods | 0.5 (1) |
| Monde Mahkota Biskuit | Indonesia | Boromon | Snacks and finger foods | 0.5 (1) |
| Products identified in National Capital Region, Philippines (n=211) | | | | |
| Happy Family Organics | USA | Happy Baby Organics | Dry or instant cereals/starch (n=3); Pureed foods (n=18); Snacks and finger foods (n=16) | 17.5 (37) |
|  |  | Happy Tot | Pureed foods (n=9); Snacks and finger foods (n=2) | 5.2 (11) |
| Nestlé | Switzerland | Cerelac | Dry or instant cereals/starch (n=8); Snacks and finger foods (n=4) | 5.7 (12) |
|  |  | Gerber | Pureed foods (n=12); Snacks and finger foods (n=7) | 9.0 (19) |
| Only Organic | New Zealand | Only Organic | Pureed foods (n=26); Snacks and finger foods (n=1) | 12.8 (27) |
| Little Freddie | United Kingdom | Little Freddie | Dry or instant cereals/starch (n=4); Pureed foods (n=13); Snacks and finger foods (n=5) | 10.4 (22) |
| Rafferty’s Garden | Australia | Rafferty’s Garden | Pureed foods | 7.6 (16) |
| The Hain Celestial Group, Inc. | USA | Earth’s Best Organic | Pureed foods (n=10); Snacks and finger foods (n=1) | 5.2 (11) |
| Alnut | Spain | Byba | Pureed foods | 3.3 (7) |
| Nutri-Del | Philippines | Nutri-Del | Dry or instant cereals/starch | 2.8 (6) |
| Organix | United Kingdom | Organix | Dry or instant cereals/starch | 2.4 (5) |
|  |  | Organix Goodies | Snacks and finger foods | 0.5 (1) |
| Bubs Australia | Australia | Bub Organic | Dry or instant cereals/starch | 2.4 (5) |
| Health Times | USA | Health Times | Snacks and finger foods | 1.9 (4) |
| Kalbe Nutritionals | Indonesia | Milna | Dry or instant cereals/starch (n=2); Snacks and finger foods (n=2) | 1.9 (4) |
| Piccolo | United Kingdom | Piccolo | Pureed foods | 1.9 (4) |
| Woolworths | Australia | Baby Macro | Pureed foods | 1.9 (4) |
| Want-Want Foods | China | Baby Mum-Mum | Snacks and finger foods | 1.4 (3) |
|  |  | Toddler Mum-mum | Snacks and finger foods | 0.5 (1) |
| Pronuben | Spain | Pronuben Baby | Pureed foods | 1.4 (3) |
| NutriDense Food Manufacturing Corp. | Philippines | Rimo | Dry or instant cereals/starch (n=1); Snacks and finger foods (n=1) | 1.0 (2) |
| Sprout Foods Inc. | USA | Sprout Foods | Snacks and finger foods | 1.0 (2) |
| Nosh Foods | USA | Baby Munchables | Snacks and finger foods | 0.9 (2) |
| Kiwigarden | New Zealand | Kiwigarden | Snacks and finger foods | 0.5 (1) |
| Rebisco | Philippines | Bibibons | Dry or instant cereals/starch | 0.5 (1) |
| H.J. Heinz Company | USA | Heinz | Pureed foods | 0.5 (1) |
| ^1^Values are presented as percentage of products per location (number of products per location) | | | | |
|  |  |  |  |  |
